# Supplementary material for: Genome-wide characterization of Salmonella Typhimurium genes required for the fitness under iron restriction
Source: BMC Genom Data. 2022 Jul 22;23:55. doi: 10.1186/s12863-022-01069-3 (PMC9308263; doi:10.1186/s12863-022-01069-3)
Supplement: Supplementary file 1 — Additional file 1. Supplementary Materials. This file contains 4 supplementary tables (Table S1 – Table S4) and 6 supplementary figures (Fig. S1 – Fig. S6). [file 12863_2022_1069_MOESM1_ESM.docx]

# **Supplementary Materials**

# **Genome-wide characterization of *Salmonella* Typhimurium genes required**

# **for the fitness under iron restriction**

### Sardar Karash^1#^ Tieshan Jiang^1^ and Young Min Kwon^1,2*^

^1^Cell and Molecular Biology Program, University of Arkansas, Fayetteville, AR 72701,

^2^Department of Poultry Science, University of Arkansas, Fayetteville, AR 72701

^#^Current address: Department of Microbiology and Immunology, University of Iowa, Iowa City, IA 52242

**Table S1. Effect of different concentrations of 2,2`-Dipyridyl (Dip) on the growth of the wild-type *S*. Typhimurium 14028s**

| **Condition** | **N** | **Growth Rate (GR)** | **Max OD_600_** | **GR % Reduction** | **Max OD_600_ % Reduction** |
| --- | --- | --- | --- | --- | --- |
| **LB** | 7 | 0.0240 | 0.938 | 0.00 | 0.00 |
| **Dip100** | 8 | 0.0227 | 0.897 | 5.37 | 4.39 |
| **Dip150** | 8 | 0.0220 | 0.834 | 8.26 | 11.00 |
| **Dip250** | 8 | 0.0201 | 0.615 | 16.30 | 34.40 |
| **Dip400** | 7 | 0.0176 | 0.488 | 26.40 | 48.00 |

**Table S2. The time required for Tn-seq selection cultures to reach the mid-log phase**

|  | **Time to reach mid-log phase** | **OD_600_** |
| --- | --- | --- |
| **Library-A (**325,000 mutants) | | |
| **LB-II** | 5 hr 0 min | 2.63 |
| **Dip100** | 5 hr 35 min | 2.61 |
| **Dip150** | 6 hr 5 min | 2.57 |
| **Library-AB (**650,000 mutants) | | |
| **LB-III** | 5 hr 30 min | 2.57 |
| **Dip250-I** | 10 hr 0 min | 2.45 |
| **Dip250-II** | 10 hr 0 min | 2.46 |
| **Dip400** | 12 hr 0 min | 1.84 |

**Table S3. The summary of the HiSeq sequencing reads**

|  | **Reads with Tn5** | **Extracted reads** | **Mapped Reads** | **Unique Insertions** |
| --- | --- | --- | --- | --- |
| **LB-II** | 38,808,640 | 31,728,005 | 25,223,444 | 125,449 |
| **Dip100** | 25,788,698 | 21,034,947 | 16,991,894 | 117,474 |
| **Dip150** | 36,677,408 | 29,905,496 | 24,364,738 | 121,132 |
| **LB-III** | 57,779,778 | 47,575,248 | 39,248,662 | 193,728 |
| **Dip250-I** | 29,832,849 | 25,082,465 | 21,096,630 | 179,562 |
| **Dip250-II** | 35,439,669 | 28,119,351 | 23,104,233 | 181,534 |
| **Dip400** | 30,028,187 | 26,382,625 | 23,135,546 | 169,666 |
| **Total** | 254,355,229 | 209,828,137 | 173,165,147 | 1,088,545 |

**Table S4. The primers used for Tn-seq library preparation**

| **Primer name** | **DNA sequence (5’→3')** |
| --- | --- |
| Tn5-DPO | AAGCTTGCATGCCTGCAGGTIIIIICTAGAGGATC |
| P5-BRX-TN5-MEO | AATGATACGGCGACCACCGAGATCTACACTCTTTCCCTACACGACGCTCTTCCGATCTNNNNAG-BARCODE-CCTAGGCGGCCTTAATTAAAGATGTGTATAAGAG |
| P7-16G | CAAGCAGAAGACGGCATACGAGCTCTTCCGATCTGGGGGGGGGGGGGGGG |

**Figure S1. *S*. Typhimurium growth response to 2,2`-Dipyridyl (Dip).** *S*. Typhimurium 14028s wild-type was grown in LB broth supplemented with a 2,2`-Dipyridyl (0, 100, 125, 150, 250, 500, 1000, 1500, or 2000 µM). The cultures were in a 96-well plate were incubated at 37°C in a Tecan Infinite 200 microplate reader. Maximum OD_600_ (Max OD_600_) was recorded after 18 hr incubation. Statistical significance was determined by unpaired two-tailed *t* test, **P* < 0.05.

**
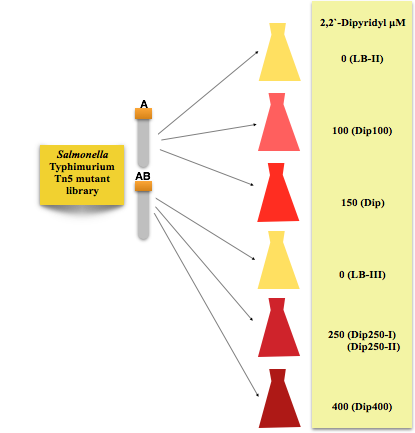
**

**Figure S2. Schematic representation of Tn-seq study design using different levels of iron restriction conditions.** Tn5 mutant libraries of *S*. Typhimurium (Library-A and Library-AB) were used to inoculate LB broth supplemented with 2,2`-Dipyridyl (Dip) at the concentrations of 100, 150, 250, and 400 µM. The controls are free of Dip. The cultures were grown until the mid- log phase. LB-II and LB-III were used as Inputs (controls) and the rest of the cultures were as Outputs for the generation and comparative analysis of the Tn-seq profiles.

**F****igure S3. Reproducibility of the Tn-seq.** Pearson correlation of the Tn-seq profiles between the two biological replicates obtained from Dip250, Dip250-I, and Dip250-II. (A) correlation between the unique insertions of Dip250-I and Dip250-II for each open reading frame (ORF). (B) correlation between the essentiality indices of Dip250-I and Dip250-II for each open reading frame (ORF).


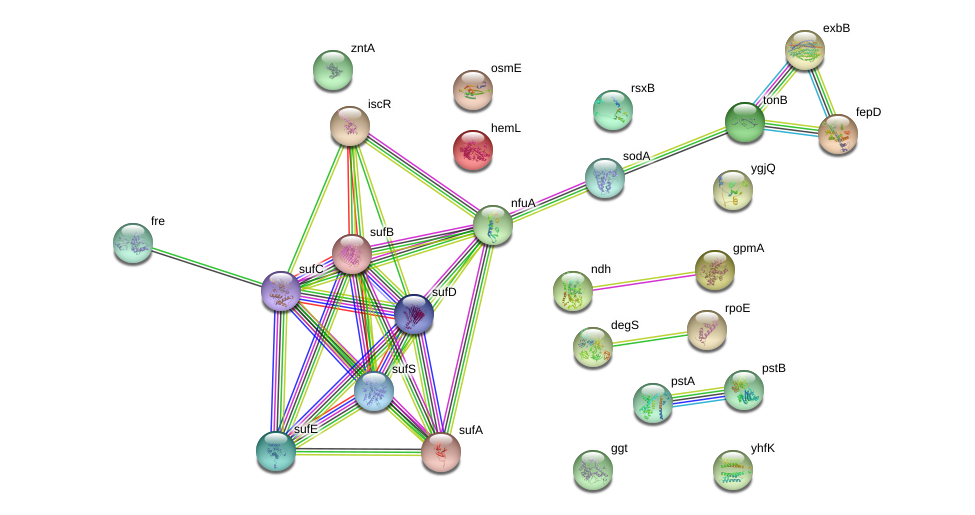


**Figure S4. Protein-protein interaction network of the genes required for the growth of *S*. Typhimurium 14028s under iron restriction conditions.** The list of the genes was as the input into String protein-protein interaction database using default options. The interactions indicate that Fe-S cluster proteins interact with siderophore proteins via NfuA and SodA.

**
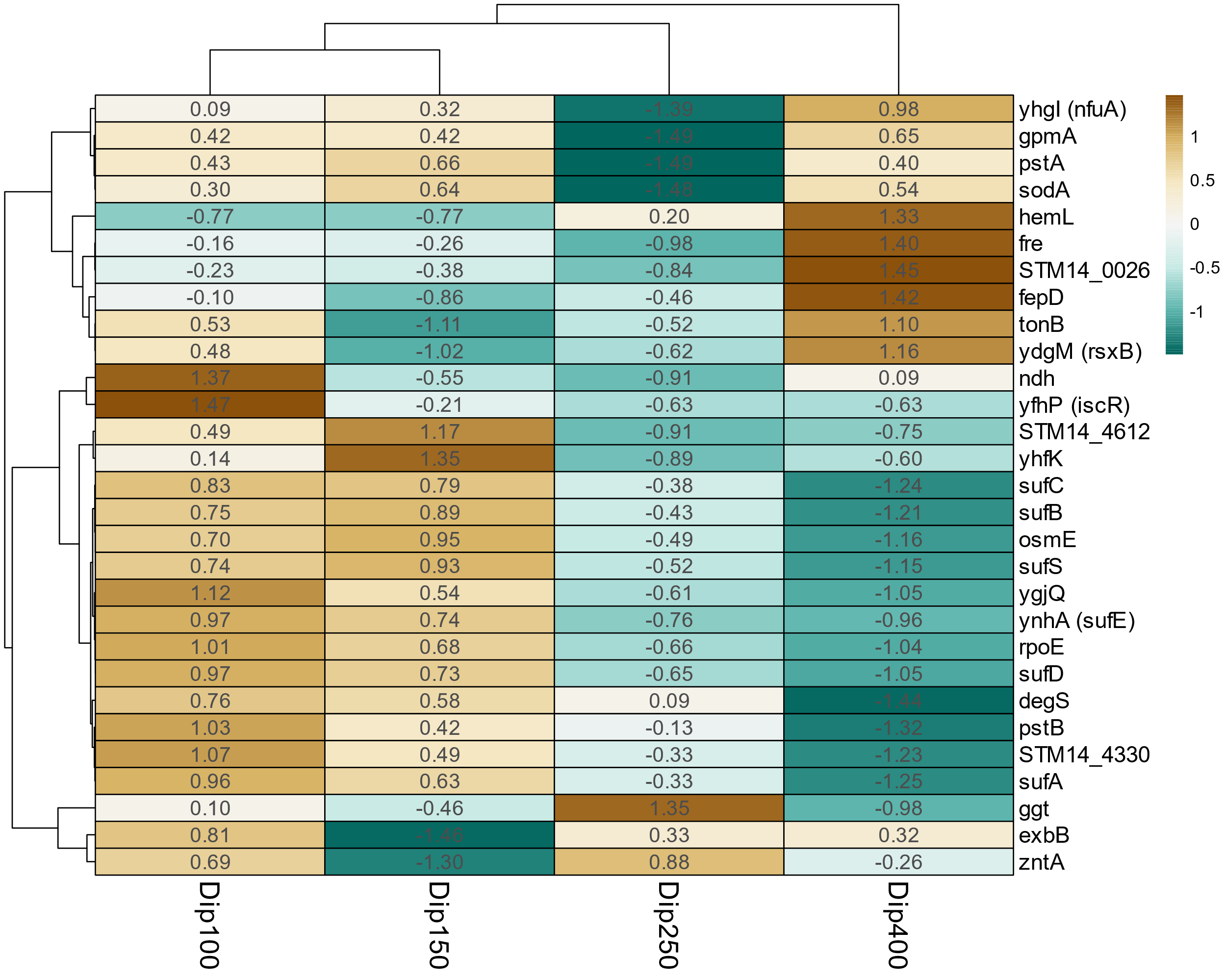
**

**Figure S5. Clustering analysis of the genes in *S.* Typhimurium 14028s identified in this study as conditionally essential for the growth under 4 different levels of iron restriction condition.** The 28 conditionally essential genes were clustered according to the changes in the fitness value of the respective mutants in response to 4 different levels (100, 150, 250, and 400 µM Dip) of iron restriction. Unit variance scaling was applied to the fitness values. Both rows and columns are clustered using correlation distance and average linkage. The clustering diagram was generated using ClustVis (biit.cs.ut.ee/clustvis).


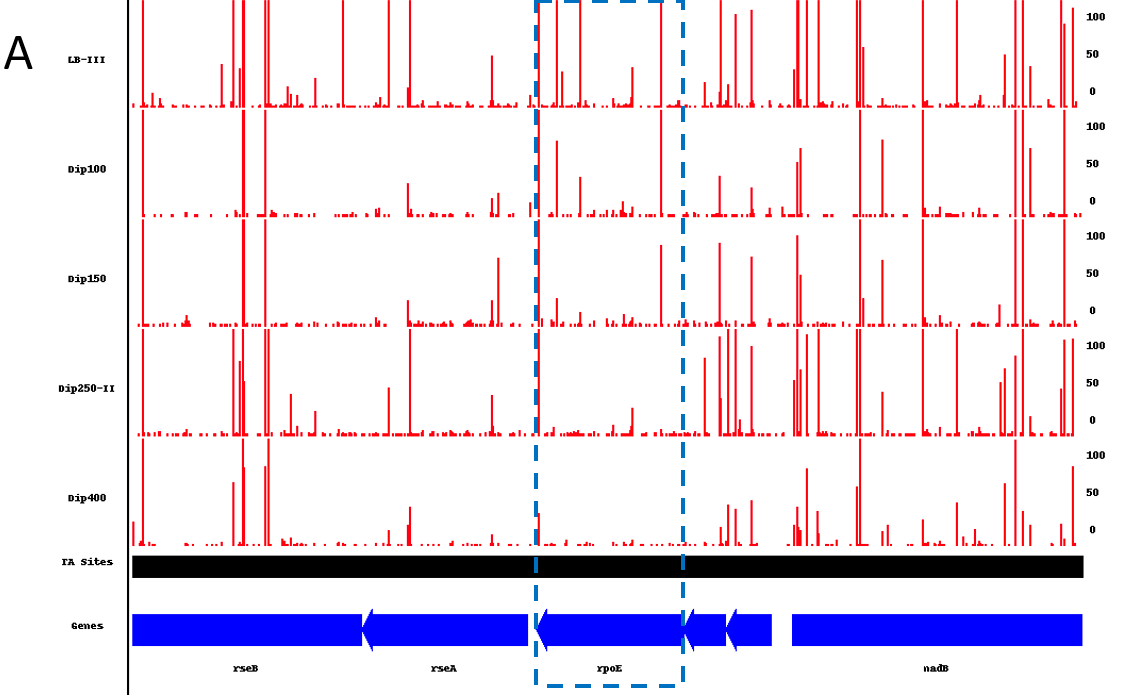


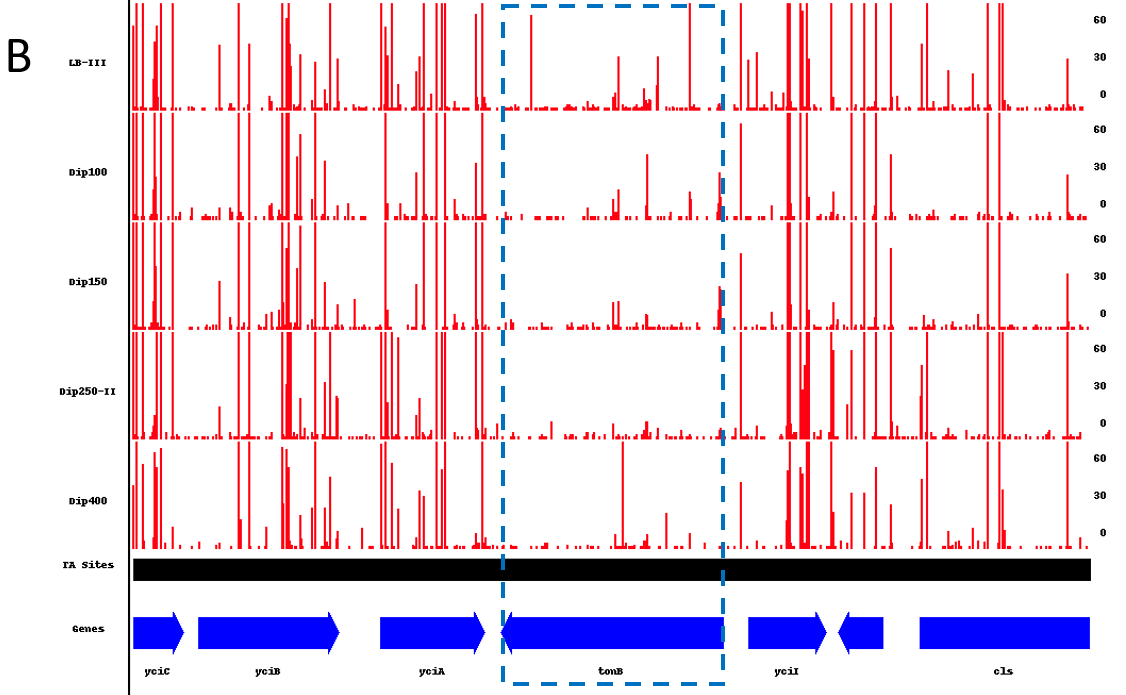


**Figure S6. Comparison of the Tn-seq profiles for (A) *rpoE* and (B) *tonB* and their surrounding genes across different levels of iron restriction (100, 150, 250, and 400 µM Dip)**
